# Site Specific Effects of Zoledronic Acid during Tibial and Mandibular Fracture Repair

**DOI:** 10.1371/journal.pone.0031771

**Published:** 2012-02-16

**Authors:** Yan Yiu Yu, Shirley Lieu, Diane Hu, Theodore Miclau, Céline Colnot

**Affiliations:** 1 Department of Orthopaedic Surgery, University of California San Francisco, San Francisco, California, United States of America; 2 INSERM U781, Hôpital Necker Enfants Malades, Paris, France; Ohio State University, United States of America

## Abstract

Numerous factors can affect skeletal regeneration, including the extent of bone injury, mechanical loading, inflammation and exogenous molecules. Bisphosphonates are anticatabolic agents that have been widely used to treat a variety of metabolic bone diseases. Zoledronate (ZA), a nitrogen-containing bisphosphonate (N-BP), is the most potent bisphosphonate among the clinically approved bisphosphonates. Cases of bisphosphonate-induced osteonecrosis of the jaw have been reported in patients receiving long term N-BP treatment. Yet, osteonecrosis does not occur in long bones. The aim of this study was to compare the effects of zoledronate on long bone and cranial bone regeneration using a previously established model of non-stabilized tibial fractures and a new model of mandibular fracture repair. Contrary to tibial fractures, which heal mainly through endochondral ossification, mandibular fractures healed via endochondral and intramembranous ossification with a lesser degree of endochondral ossification compared to tibial fractures. In the tibia, ZA reduced callus and cartilage formation during the early stages of repair. In parallel, we found a delay in cartilage hypertrophy and a decrease in angiogenesis during the soft callus phase of repair. During later stages of repair, ZA delayed callus, cartilage and bone remodeling. In the mandible, ZA delayed callus, cartilage and bone remodeling in correlation with a decrease in osteoclast number during the soft and hard callus phases of repair. These results reveal a more profound impact of ZA on cartilage and bone remodeling in the mandible compared to the tibia. This may predispose mandible bone to adverse effects of ZA in disease conditions. These results also imply that therapeutic effects of ZA may need to be optimized using time and dose-specific treatments in cranial versus long bones.

## Introduction

Bisphosphonates (BP) are synthetic analogs of pyrophosphate that can be incorporated in vivo into mineralized tissues [1]. Due to their potent effects on osteoclastic bone resorption, BP are widely used for the treatment and/or prevention of metabolic bone diseases characterized by increased osteoclast activity such as Paget's disease, metastatic and osteolytic bone diseases, as well as osteoporosis [2]. These anti-resorptive effects of BP are also explored for improvement of callus strength and fracture healing in combination with anabolic treatments [3]. There are two major classes of bisphosphonates. The first group contains the less potent, non-nitrogen containing BP that can be metabolized into nonhydrolyzable analogues of ATP [4] and the second group contains the more potent, nitrogen containing BP such as alendronate, risedronate, and zoledronate (ZA). These potent BPs interfere with the mevalonate biosynthetic pathway and inhibit protein prenylation, which is important for osteoclast function [2], [5]. In 2002, intravenous ZA was approved to treat patients with multiple myeloma and bone metastases. Although ZA is the most effective BP in clinical use, undesirable effects have been reported that require better understanding of its mechanisms of action in bone. Cases of osteonecrosis of the jaw (ONJ) have been reported in patients treated with high dose of nitrogen containing bisphosphonates [6]–[8], but no reports were found in long bones. As ONJ is often associated with implant procedures, the bone repair process occurring around the implant is considered a key event leading to bone necrosis. The adverse effects of ZA could result from differences in mandibular and long bone repair. Bones in the head and the appendicular skeleton are derived from distinct cell lineages during embryonic development, which may lead to differences in their regenerative capacities and susceptibility to bisphosphonate treatment [9]–[11]. The goal of this study was to contrast the impact of ZA on the repair of mandibular and tibial fractures. We used a well-established model of non-stabilized tibial fracture and created a mouse mandibular fracture model to evaluate the effects of ZA on cranial versus long bone repair at the cellular and molecular levels. Bisphosphonates may not only act on osteoclast function but also on other cell types that are required for a timely repair process including osteoblasts, chondrocytes and endothelial cells [12], [13]. Therefore, we assessed the consequences of ZA treatment on cartilage and bone formation within the fracture callus, as well as matrix remodeling, osteoclasts and angiogenesis.

## Materials and Methods

### Zoledronate Treatment

All procedures followed protocols approved by the UCSF Animal Care and Use Committee (approval number AN080353-02B). Adult C57B6 wild type mice (males 3–4 month old) were anesthetized with an intraperitoneal injection of 50 mg/ml Ketamine/0.5 mg/ml Metedomidine (0.03 ml/mouse) and received 3 µg (0.1 mg/kg) Zoledronate (ZA) (Zometa, Novartis Pharma AG, Basel Switzerland) in 200 µl saline intravenously once 4 weeks before fracture and once at the time of fracture. This dosage is based on that which is typically used for treatment of multiple myeloma or breast cancer in humans [7], [14]. Control mice received the same volume of saline solution intravenously.

### Non-stabilized tibial fractures

**Figure 1:**
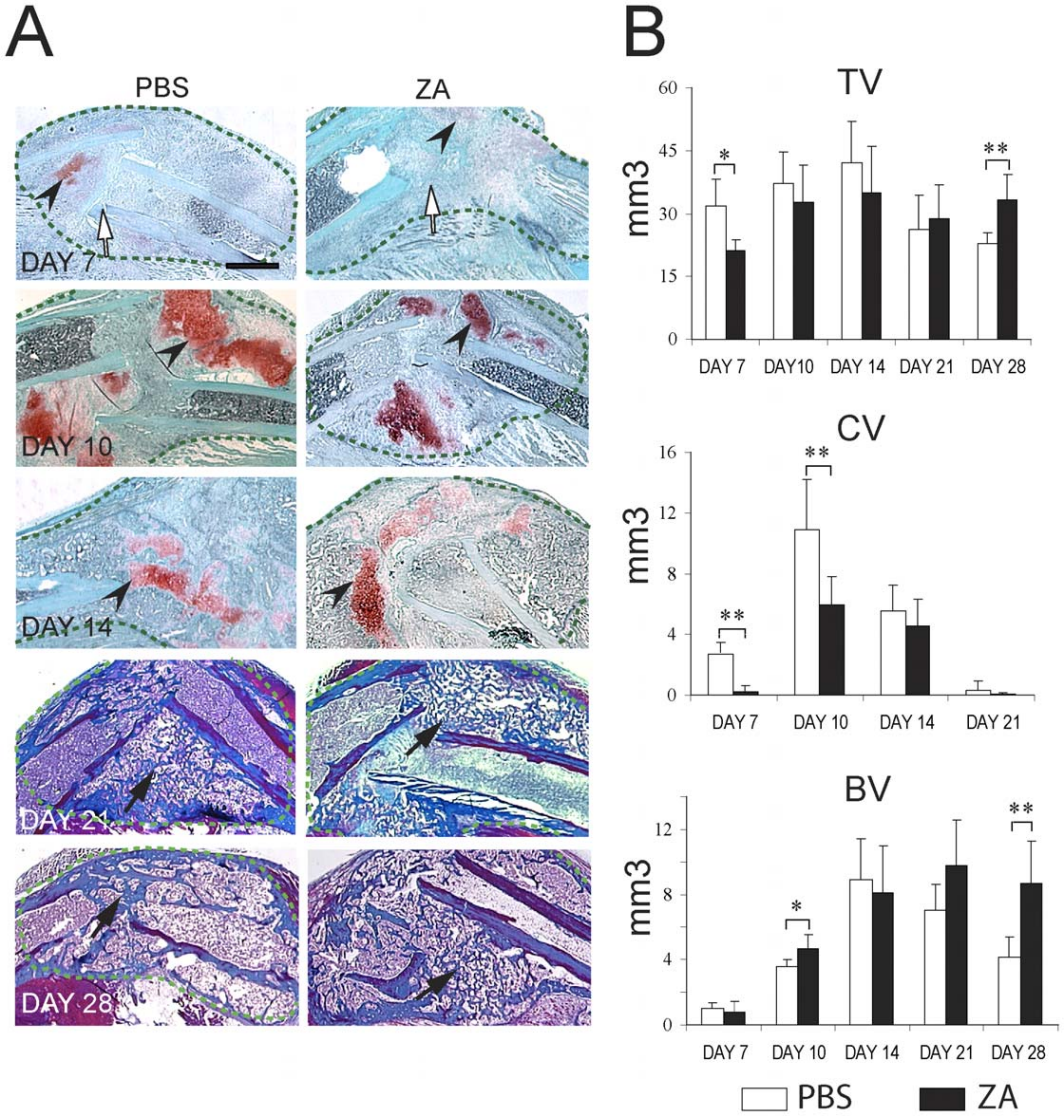
Effects of Zoledronate on the course of tibial fracture repair. (A) Sections through the calluses (green dashed-lines) of PBS- (left) and zoledronate- (right) treated mice were stained with Safranin-O/Fast Green (days 7, 10 and 14 post-fracture) to detect cartilage (arrowsheads) and Trichrome (days 21 and 28 post-fracture) to detect bone (arrows). White arrows indicate the fracture site. Scale bar = 500 µm. (B) Histomorphometric analyses of total callus volume (Top), total cartilage volume (CV, middle) and total bone volume (BV, bottom) on PBS treated and ZA treated-mice at days 7, 10, 14, 21 and 28 post-fracture (n = 6 per group). *p<0.05, **<0.01. Bars represent mean±s.d.

Adult C57B6 wild type mice (males 3–4 month old) were anesthetized with an intraperitoneal injection of 50 mg/ml Ketamine/0.5 mg/ml Medetomidine (0.03 ml/mouse). Closed, standardized non-stabilized fractures were produced in the mid-diaphysis of the right tibia via three point bending as previously described [15] (Fig. 1). Mice were sacrificed by cervical dislocation following an intraperitoneal injection of 2% Avertin (0.5 ml/mouse) and fracture calluses were collected at 5, 7, 10, 14, 21 and 28 days post-fracture (n = 5 per group). Mandibular and tibial fractures were induced in two separate groups of animals.

### Non-stabilized mandibular fractures

**Figure 2:**
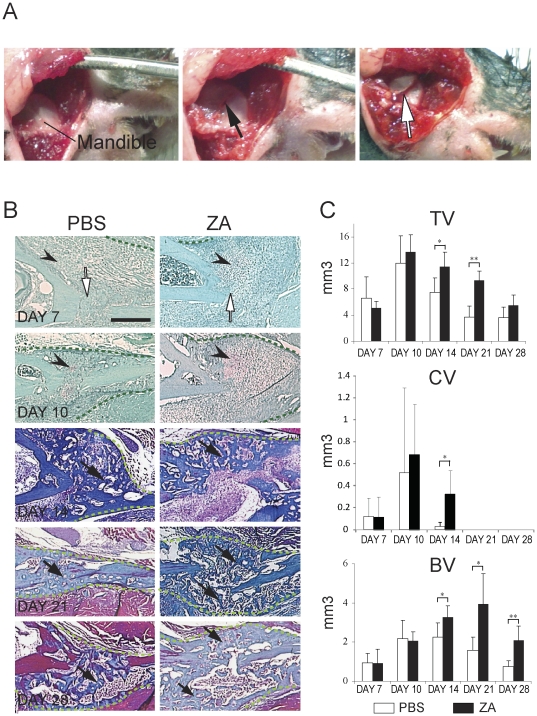
Mandibular fracture model and effects of zoledronate on the course of mandibular fracture repair. (A) To create a mandibular fracture, the skin was opened and the mandible was exposed (left). Five holes were drilled (black arrow) and the fracture was created (white arrow) by surgical tweezers. (B) Sections through the PBS- (left) and zoledronate- (right) treated calluses (green dashed-lines) were stained with Safranin-O/Fast Green (day 7 and day 10 post-fracture) to detect cartilage (arrowheads) and Trichrome (day 14, 21, and 28 post-fracture) to detect bone (arrows). White arrows indicate the fracture site. Scale bar = 500 µm. (C) Histomorphometric analyses of total callus volume (TV, Top), total cartilage volume (CV, middle) and total bone volume (BV, bottom) on PBS treated and zoledronate treated-mice at days 7, 10, 14, 21 and 28 post-fracture (n = 6 per group). *p<0.05, **<0.01. Bars represent mean±s.d.

Adult C57B6 wild type mice (males 3–4 month old) were anesthetized with an intraperitoneal injection of 50 mg/ml Ketamine/0.5 mg/ml Medetomidine (0.03 ml/mouse) and received a preoperative dose of antibiotic (cefazolin, 10 mg/kg). For non-stabilized mandibular fractures, an incision was made along the inferior portion of the right hemimandible, and the masseter muscle was divided along its length and elevated to expose the body of the mandible bone. Five holes were created using a high-speed dental drill filled with an insect-pin along the mandible bone from the coronoid process to the gonial angle of the mandible bone (Fig. 2). Fracture was created using surgical tweezers. After creating the fracture, the soft tissues and skin were closed and animals were given analgesics. All animals were fed with soft diet for at least 7 days. All animals received a subcutaneous injection of buprenorphine (1.0 mg/kg) for analgesia immediately after surgery, and 4 and 24 hours after surgery. Food intake and animal activity were monitored frequently. Mice were sacrificed by cervical dislocation following an intraperitoneal injection of 2% Avertin (0.5 ml/mouse) and fracture calluses were collected at 5, 7, 10, 14, 21 and 28 days post-fracture (n = 5 per group).

### Histological and histomorphometric analyses of cartilage and bone

Callus tissues were fixed overnight at 4°C in 4% paraformaldehyde, decalcified at 4°C in 19% EDTA (pH 7.4) for 10–14 days, then dehydrated in a graded ethanol series and embedded in paraffin. Serial sections (10 µm thick) were collected throughout the entire callus and analyzed by histomorphometry as previously described [15]–[17]. To determine the amount of cartilage within each callus, every thirtieth section was stained with Safranin-O/Fast Green. To determine the amount of bone, adjacent sections were stained with Milligan's Trichrome. Images were captured from a Leica DM 5000 B light microscope (Leica Microsystems GmbH) that was equipped with a camera (Diagnostic). The area of callus, cartilage and bone was determined using Adobe PhotoShop.

### Immunohistochemistry

Representative sections were processed for immmunohistochemistry. Briefly, after deparaffinization and hydration, antigen retrieval was performed by incubating with 0.05% trypsin and followed by 3% H_2_O_2_ in methanol for 15 min to inhibit endogenous peroxidase activity. Slides were treated with 1.5% donkey serum or 1.5% goat serum as indicated by manufacturer's instruction (goat or rabbit ABC staining kit; Santa Cruz Biotechnology, Santa Cruz, CA). Sections were incubated with the primary antibody (affinity purified goat polyclonal antibody against Collagen type II, Santa Cruz Biotechnology, CA; affinity purified rabbit polyclonal antibody against Collagen type X, Chemicon, Millipore, Billerica, MA) diluted (1∶50) in the blocking serum in a humidified chamber at 4°C overnight. Control sections were incubated with normal goat IgG or normal rabbit IgG serums. Detection of primary antibody binding was done using goat or rabbit ABC staining kit (Santa Cruz Biotechnology, Santa Cruz, CA). Sections were developed with DAB and counterstained with 1% Fast Green.

### Detection and quantification of osteoclasts in the fracture callus

Tartrate-resistant acid phosphatase (TRAP) staining was performed using a leukocyte acid phosphatase kit (Sigma) on serial sections (10 µm) that were 300 µm apart through the callus. The number of osteoclasts within the fracture callus was estimated using an Olympus CAST system (Olympus) and software by Visiopharm (Visiopharm) as previously described [18]. Briefly, fracture callus was outlined using low magnification in each section. Ten to twenty fields that covered approximately 10% of the fracture callus were systematically acquired using unbiased uniform random sampling under higher magnification. A counting frame probe was also selected to quantify the number of osteoclasts and the area of the callus within each field. Values are expressed as number of osteoclasts per mm^2^ callus tissue.

### Detection and quantification of apoptotic cells and osteoclasts in the fracture callus

To detect osteoclasts and apoptotic cells, TUNEL assay to detect DNA fragmentation was performed using a TUNEL kit (ApopTag, Millipore) according to the manufacturer's instructions followed by TRAP staining. The number of apoptotic cells and osteoclasts within the fracture callus was estimated on three center sections using an Olympus CAST system (Olympus) and software by Visiopharm (Visiopharm) as described above.

### Detection and quantification of tissue vascularity in the fracture callus

To visualize and quantify blood vessels at day 5 post-fracture, calluses were cryo-embedded in OCT. Serial sections (10 µm) that were 600 µm apart were selected throughout the callus and immunohistochemistry using an anti-platelet endothelial cell adhesion molecules (PECAM) antibody was performed [15]. For each sample, six sections were analyzed. The length density (length of blood vessels per unit volume of the reference space) of the blood vessels within the fracture callus and the surface density (area of the outer surface of blood vessels per unit volume of the reference space) of the blood vessels within the fracture callus were estimated using an Olympus CAST system (Olympus) and Visiopharm software (Visioplarm) as previously described [18], [19]. The whole callus was used as the reference space and its volume was estimated using Calvalieri's Principle [20], [21].

### Statistical analyses

The student t-test was used to compare experimental and control samples, and p-values of <0.05 were considered significant.

## Results

### Zoledronic Acid affects the formation and remodeling of cartilage and bone during tibial fracture repair

To examine the long-term effects of ZA on healing of non-stabilized tibial fractures, we injected ZA intravenously 4 weeks before fracture and on the day of fracture. We chose a high dose of ZA as used in cancer patients and delivered two doses in order to maximize the incorporation of ZA within bone prior to injury [7], [14]. Histological analyses showed a delay in fracture repair in ZA-treated mice (Fig. 1A). There was less cartilage during the soft callus phase of repair (day 7 to 10 post-fracture) in ZA-treated compared to PBS-treated mice as shown by Safranin-O staining (Fig. 1A). Trichrome staining did not reveal an apparent delay in bone deposition within the callus until day 14 (data not shown). However, there was more woven bone in the callus of ZA treated mice by day 28, indicating a delay in the restoration of the bone marrow cavity (Fig. 1A). Histomorphometric analyses showed that total callus volume was smaller in ZA-treated samples compared to controls at day 7. Total callus volume gradually increased from day 7 to 14 and was reduced by day 28 post-fracture in control samples, while it remained high by day 28 in ZA-treated samples indicating a delay in callus formation and remodeling (Fig. 1B, top). Histomorphometric analyses confirmed that there was significantly less cartilage in the callus of ZA-treated samples at days 7 and 10 post-fracture (Fig. 1B, middle). Cartilage volume peaked by day 10 and decreased by day 14 in PBS-treated samples, while cartilage volume remained high by day 14 compared to day 10 in ZA-treated samples (Fig. 1B, middle). These results indicate that ZA delays both cartilage deposition and remodeling during tibial fracture repair. Bone volume was significantly higher by day 10 post-fracture and remained high by day 28 in ZA-treated samples compared to controls implying an increase in bone deposition during the soft callus phase of repair followed by a delay in bone remodeling (Fig. 1B, bottom).

### Zoledronic Acid delays cartilage and bone remodeling during non-stabilized mandibular fracture repair

To compare the effects of ZA on tibial and mandibular fracture repair, we developed a non-stabilized mandibular fracture model. Fractures were created from the top of the coronoid process to the gonial angle of mandible bone (Fig. 2A). Histological analyses showed that mandibular fractures healed via endochondral ossification with a lesser degree of cartilage deposition compared to non-stabilized tibial fractures (Fig. 2B). Callus size was smaller in mandibular compared to tibial fractures. As observed in tibial fractures, mandible fracture calluses were filled with woven bone by day 14 post-fracture, and gradually resorbed and bridged by day 21. Bone remodeling was well underway by day 28 (Fig. 2B, left). Histomorphometric analyses confirmed these histological analyses. Total callus volume peaked at day 10 and gradually reduced by day 28 post-fracture in PBS- and ZA-treated samples, but the total callus volume was significantly greater in ZA-treated samples compared to the controls at days 14 and 21 post-fracture indicating that there was a delay in callus remodeling (Fig. 2C, top). Cartilage formed by day 7, peaked at day 10 and decreased by day 14, but cartilage volume remained significantly high in ZA-treated calluses compared to PBS-treated calluses. Cartilage was completely resorbed by day 21 in both ZA- and PBS-treated calluses (Fig. 2C, middle). Bone volume gradually increased from day 7 to day 14 and decreased by day 28 in PBS-treated calluses, but remained high from day 14 to day 28 in ZA-treated calluses (Fig. 2C, bottom). These data indicate a delay in cartilage and bone remodeling in the repair of non-stabilized mandible fractures treated with ZA.

### Zoledronic acid delays cartilage hypertrophy during tibial fracture healing

**Figure 3:**
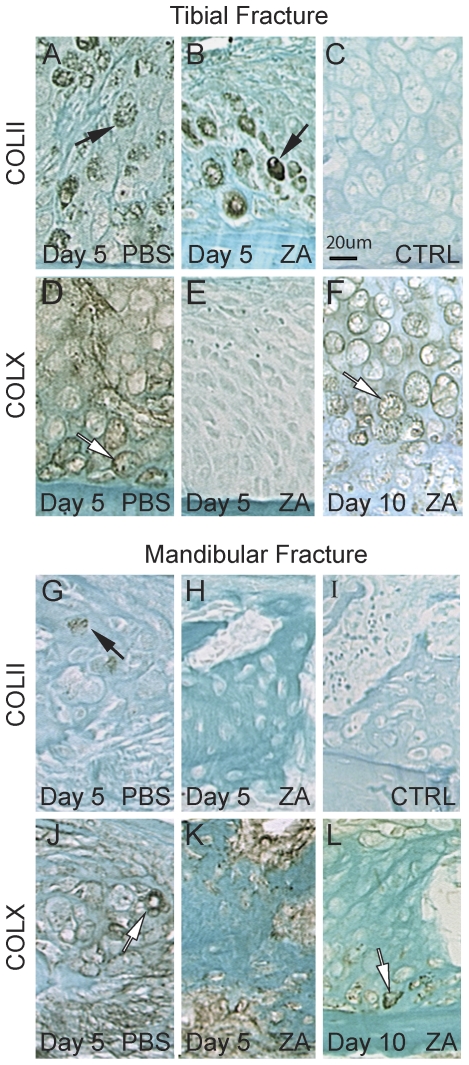
Effects of Zoledronate on collagen types II and X expression during tibial and mandibular fracture repair. Immunostaining of collagen type II (A–B, G–H, black arrows) and collagen type X (D–F, J–L, white arrows) near the periosteum within tibial (top) and mandibular (bottom) fracture calluses of PBS (left) and zoledronate (middle and right) treated mice. No staining is observed in negative controls (C and I).

Since cartilage formation was delayed in tibial but not in mandibular fracture healing, we performed immunohistochemistry to examine chondrocyte differentiation following ZA and PBS treatment. In tibial fracture calluses, immunostaining for collagen type II was detected in chondrocytes of both PBS and ZA-treated mice by day 5 post-injury (Fig. 3A, B). However, collagen type X immunostaining was observed in PBS but not in ZA-treated calluses (Fig. 3D, E). By day 10, collagen type X was expressed in both groups (Fig. 3F and data not shown), therefore cartilage hypertrophy was delayed during tibial fracture repair as a result of ZA treatment. In mandibular fracture calluses, immunostaining for collagen type II was markedly decreased compared to tibial fractures (Fig. 3G, H), which was in concordance with decreased cartilage formation reported via histology and histomorphometry. Immunostaining for collagen type X was seen in the calluses of PBS and ZA-treated mice at days 5 and 10 post-fracture (Fig. 3J, K, L and data not shown).

### Zoledronic acid reduces osteoclastogenesis during mandibular fracture healing

**Figure 4:**
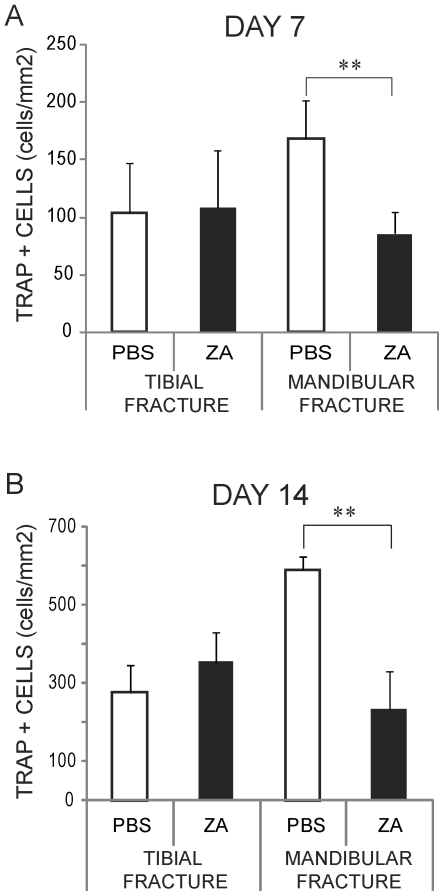
Effects of zoledronate on osteoclastogenesis during tibial and mandibular fracture repair. Stereological analyses of TRAP positive cells per area of fracture callus (n = 6 per group) in PBS- and zoledronate-treated mice at day 7 (A) and day 14 (B) post-fracture. **p<0.01. Bars represent mean±s.d.

**Figure 5:**
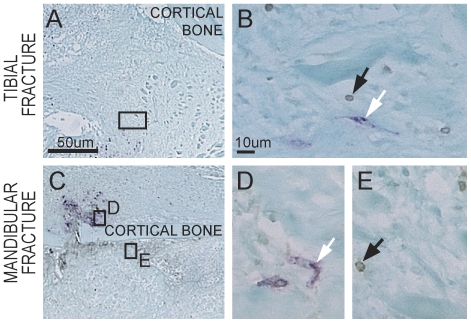
Effects of zoledronate on apoptosis during tibial and mandibular fracture repair. TUNEL and TRAP double staining on ZA-treated tibial (A and B) and mandibular (C–E) fractures at day 5 post-injury. High magnifications (B, D, E) indicate apoptotic cells (black arrows) and TRAP-positive cells (white arrows) within the fracture callus. No TRAP-positive apoptotic cells can be detected at the fracture site. (A, C) Scale bar = 50 µm; (B, D, E) Scale bar = 10 µm.

Callus formation and remodeling involves the deposition and breakdown of extracellular matrix by the coordinated action of osteoblasts, chondrocytes and osteoclasts. Osteoclasts are a well-known target of bisphosphonates action. Therefore, we investigated the effects of ZA on osteoclast numbers in tibial and mandibular fractures. There were no significant differences in the number of TRAP-positive cells per callus area between ZA-treated and control samples in tibial fractures, but there were less TRAP-positive cells per callus area in ZA-treated samples compared to the controls in mandibular fractures at both days 7 and 14 post-fracture (Fig. 4). To assess whether the decrease in the number of TRAP-positive cells was due to apoptosis, we performed double staining for TUNEL and TRAP at day 5 post-fracture. Overall there was minimal cell death in the callus of both tibial and mandibular fractures, and we did not observe significant differences between ZA-treated and control groups (data not shown). Dead cells were found at the fracture site, within the bone marrow and in the cortex, while TRAP-positive cells were found primarily around new bone adjacent to the periosteum and within the fracture callus (Fig. 5 and data not shown). Double staining and quantitative analyses showed that TRAP-positive cells were not TUNEL-positive, suggesting that ZA treatment did not induce osteoclast cell death (Fig. 5 and data not shown). Therefore, the decrease in the number of TRAP-positive cells were not due to increase cell death in mandibular fractures.

### Zoledronic acid reduces angiogenesis in the tibial fracture callus

**Figure 6:**
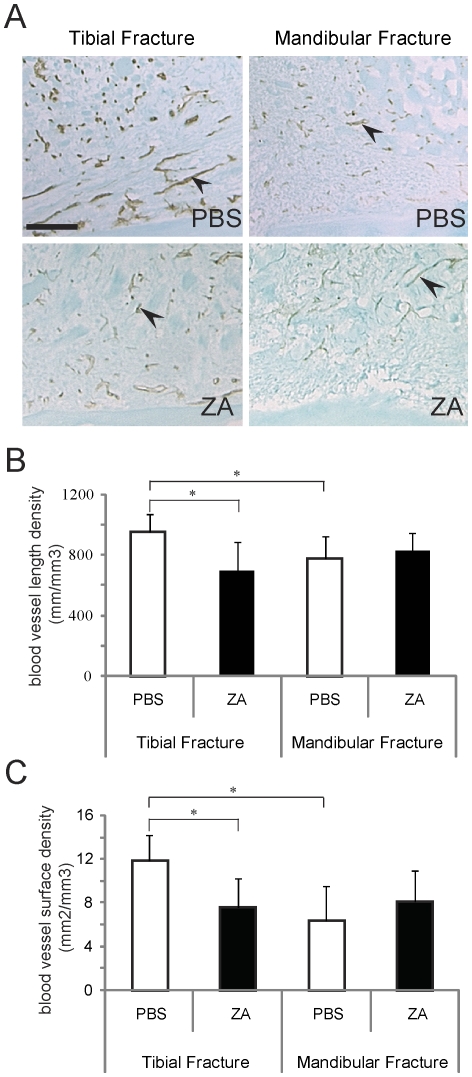
Effects of zoledronate on angiogenesis during tibial and mandibular fracture repair. (A) PECAM immunostaining (black arrows) on PBS- (top) and ZA-treated (bottom) tibial (left) and mandibular (right) fracture calluses. Scale bar = 200 µm. (B) Analysis of the length density of blood vessels within the callus (mm/mm^3^) and (C) analysis of the surface density of blood vessels within the callus (mm^2^/mm^3^) in PBS- and zoledronate-treated mice at day 5 post-fracture (n = 6 per group). *p<0.05. Bars represent mean±s.d.

Skeletal tissue deposition and remodeling is tightly linked with revascularization of the fracture site. To examine whether ZA affects vascularization, we quantified blood vessel length and surface densities in ZA- and PBS-treated calluses. Immunohistochemistry of PECAM (Fig. 6A) and stereological analyses revealed that blood vessel length density (Fig. 6B) and surface density (Fig. 6C) were significantly reduced in ZA-treated calluses compared to controls in tibial fractures but not in mandibular fractures at day 5 post-fracture. Our results also revealed that in the absence of ZA treatment, blood vessel length density and surface density were significantly higher in tibial fractures compared to mandibular fractures (Fig. 6).

## Discussion

### Differences between tibial and mandibular fracture repair

In this study, we investigated the differences in healing between cranial and long bones and the responses to ZA treatment in these two repair sites. We created a non-stabilized mandibular fracture model to compare healing with our previously described model of non-stabilized tibial fractures [15], [22]. While non-stabilized tibial fractures heal mostly through endochondral ossification, we show that non-stabilized mandibular fractures heal through intramembranous and endochondral ossification with a reduced amount of endochondral ossification compared to tibial fractures. Differences between tibial and mandibular fracture repair may be due to differences in their mechanical environment [22], [23]. Previous studies in our laboratory showed that tibial fractures heal via intramembranous ossification when a rigid fixation is applied to the fracture site, thus indicating that the mechanical environment influences the mode of repair [15], [22]. In the absence of mechanical stimuli, mesenchymal cells differentiate directly into osteoblasts but under the influence of mechanical stimuli cells can differentiate into osteoblasts and chondrocytes [22]. Mechanical stimuli also play a significant role in bone repair by regulating vascular growth [24], [25]. VEGF and TGF-beta are released from human fracture hematoma with mechanical loading [24]. In our study both tibias and mandibles were loaded. However, the mechanical stimuli and stress are presumably greater in the tibia due to weight bearing, which may explain the increase in callus size, number of blood vessels and degree of endochondral ossification compared to mandibular fractures.

Differences in the extent of endochondral ossification between tibial and mandibular fracture calluses may also be due to the distinct cell populations that participate in repair. Bones in the head and the trunk do not share the same embryonic origins. Long bones in the limb are derived from the trunk lateral plate mesoderm and form via endochondral ossification, while the mandible is derived from the paraxial mesoderm in the head, and forms via intramembranous ossification [10]. We previously showed that skeletal progenitor cells are recruited from the local periosteum during long bone repair [26]. Other reports indicate that cells in the mandible are recruited locally during repair [27]. Given the distinct embryonic origins of cells that constitute the mandible and the tibia, progenitor cells in the mandible may be more prone to undergo bone formation via intramembranous ossification while cells in the tibia may be more prone to undergo endochondral ossification. Yet it is interesting to note that both tibia and mandible can form bone via endochondral ossification in a non-stable mechanical environment and via intramembranous ossification in a stable mechanical environment. Regardless of the source of cells, the mechanical environment largely influences cell fate after bone injury. These fundamental differences between tibial and mandibular fractures may in part explain the differential effects of ZA in these two healing environments. On the one hand skeletal progenitors within cranial and long bones may not have the same sensitivity to ZA, thus leading to direct effects on osteoblasts and/or chondrocytes. On the other hand, indirect effects of ZA may be due to the distinct mechanical environments and effects on other cell types such as osteoclasts and endothelial cells.

### Site-specific effects of Zoledronic Acid in late stages of fracture repair

Although ZA does not prevent fracture healing, we observed significant changes during healing of mandible and tibial fractures as a consequence of ZA treatment. This was not due to an insufficient dosage of ZA since we maximized the dosage based on that used in cancer therapies [7], [14]. We show that intravenous ZA injection increases the callus size and bone volume during the remodeling phase of both mandibular and tibial fracture repair. This is consistent with previous studies, which reported that ZA treatment delays bone remodeling and simultaneously increases the strength of the callus during late stages of fracture repair [28]. In addition, we report a delay in cartilage remodeling, suggesting that ZA affects matrix-remodeling in both cartilage and bone. Interestingly, we observed a more severe remodeling defect in mandibular compared to tibial fractures, which was linked to a decrease in the number of osteoclasts in the ZA treated mandibles. This decrease in osteoclast number was not due to increased cell death, and may therefore result from a decrease in the infiltration of osteoclasts in the callus [29]–[31]. This more pronounced remodeling defect in the mandible may also result from impaired osteoclast function [30]. Compared to long bones, osteoclasts in cranial bones exhibit higher levels of proteolytic enzymes and resorptive activities, which may augment their sensitivity to ZA treatment [32], [33].

### Site-specific effects of Zoledronic Acid in early stages of fracture repair

Previous studies on the effect of bisphosphonates on fracture healing mostly focused on later stages of repair, where they induce a delay in callus remodeling both in stabilized and non-stabilized mechanical environments [34]–[36]. Our study reveals the effects of ZA in the early stages of bone repair and the process of endochondral ossification. We treated mice starting one month prior to the injury in order to specifically assess long-term effects of ZA and the impact on the initial stages of repair. There was a decrease in callus size and cartilage volume in the early soft callus phase of tibial fracture repair, but this effect was not seen in mandibular fractures. In parallel, ZA delayed chondrocyte hypertrophy in tibial but not mandibular fractures. A direct effect of ZA on chondrogenesis may be more apparent in tibial factures due to increased cartilage volume and higher degree of endochondral ossification. Concurrently, we detected a decrease in angiogenesis in tibial fractures treated with ZA compared to control groups. Impaired angiogenesis could result from delayed hypertrophy and delayed VEGF expression in cartilage. Several studies reported that bisphosphonates interfere with the angiogenic process through various molecular pathways [37]–[41]. This delay in re-vascularisation of the fracture site might also affect cartilage formation. Indeed, we have previously observed a correlation between low blood vessel density and inhibition of chondrogenesis during the initial phase of stabilized fracture repair [19]. In parallel, we observed early anabolic effects on bone, which can be attributed to stimulatory effects on osteoblast proliferation in vitro [42]–[45].

In conclusion, we report differential effects of ZA on cranial versus long bone repair in mice. The soft callus phase of repair was more affected in the tibia compared to the mandible with a delay in cartilage and bone deposition, in cartilage hypertrophy and angiogenesis. ZA had a more profound impact on tissue remodeling in mandibular compared to tibial fractures, which was correlated with a decrease in osteoclasts. These site-specific effects of ZA do not necessarily explain the occurrence of ONJ, since other factors are involved such as infection, combined treatment with corticosteroids, immunosuppressive agents or chemotherapy [46]–[48]. These results suggest that BP can be used as anticatabolic agents to augment bone repair with or without combination with anabolic agents [3], [28], however, the dosage and the timing of anti-resorptive therapy to augment the size and strength of the callus may change depending on the type of bone and the mechanical environment.
